# P-1680. Performance of direct Disk Diffusion (dDD) testing in gram negative rod bacteremia as a tool for early escalation of therapy

**DOI:** 10.1093/ofid/ofaf695.1854

**Published:** 2026-01-11

**Authors:** Kathryn DeSear, Kenneth Rand, Tori Gray

**Affiliations:** University of Florida College of Pharmacy, Gainesville, FL; University of Florida, Gainesville, Florida; UF Health Shands Hospital, Cincinnati, Ohio

## Abstract

**Background:**

In 2021, CLSI endorsed a novel approach for preliminary susceptibility testing - direct Disk Diffusion (dDD) which involves applying antibiotic disks directly to fluid from the positive blood culture bottles, using non-standardized inocula. By providing preliminary susceptibility results within 8–24 hours of the growth signal, dDD offers a significantly faster alternative to traditional AST.

This study aims to evaluate the performance and reliability of dDD in detecting antimicrobial susceptibility in *Enterobacterales* and *Pseudomonas aeruginosa*. The primary outcome measure is positive predictive value (PPV), which assesses the test’s accuracy in identifying isolates as susceptible when they are truly susceptible. Secondary outcomes include negative predictive value (NPV), sensitivity, specificity, major errors, and very major errors.

**Figure:**
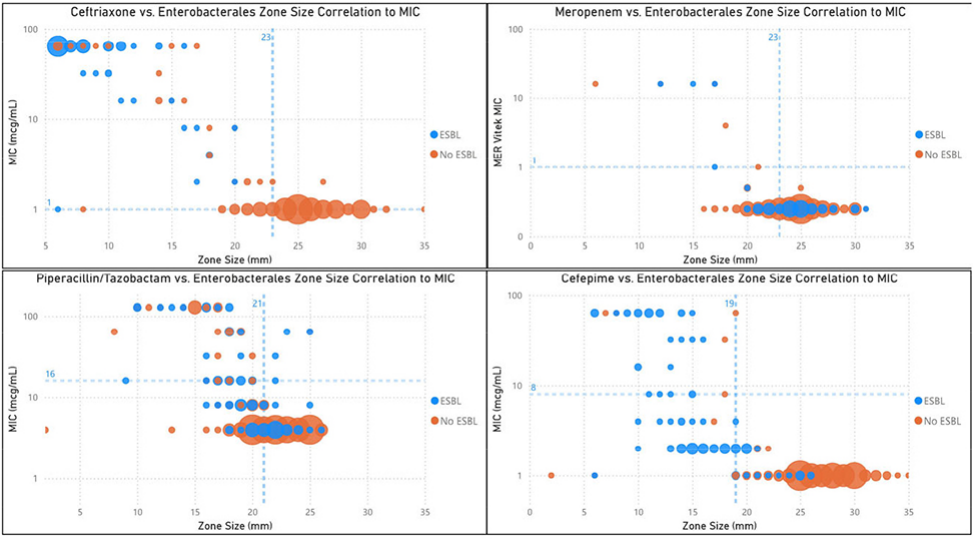
Beta Lactam Zone size correlation to MIC in Enterobacterales In each graph, a dotted line runs through the y axis at the MIC breakpoint (AST) and a dotted line runs through the x axis at the zone size cutoff (dDD). The upper left quadrant represents the true negative (resistant by dDD and AST) isolates and the bottom right quadrant represents the true positives (susceptible by dDD and AST). The top right quadrant represents the very major errors (susceptible by dDD and resistant by AST) while the bottom left quadrant represents the major errors (resistant by dDD and susceptible by AST).

**Figure:**
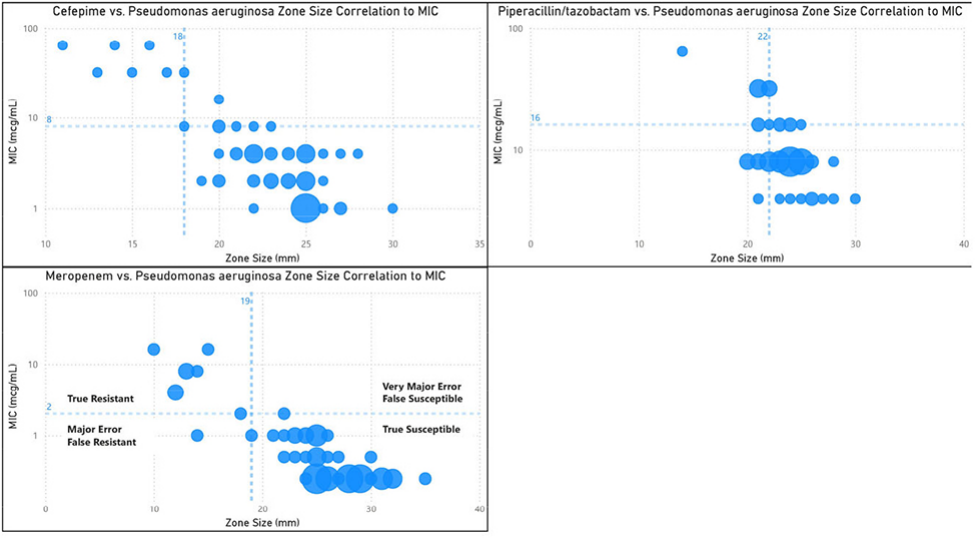
Beta lactam antibiotics Zone size correlation to MIC in Pseudomonas aeruginosa In each graph, a dotted line runs through the y axis at the MIC breakpoint (AST) and a dotted line runs through the x axis at the zone size cutoff (dDD). The upper left quadrant represents the true negative (resistant by dDD and AST) isolates and the bottom right quadrant represents the true positives (susceptible by dDD and AST). The top right quadrant represents the very major errors (susceptible by dDD and resistant by AST) while the bottom left quadrant represents the major errors (resistant by dDD and susceptible by AST).

**Methods:**

This single center retrospective cohort study included all patients with at least one positive blood culture showing gram negative bacilli on Gram’s stain. Patients were excluded if death or discharge occurred within 48 hours of blood culture draw, incomplete susceptibility results, polymicrobial bacteremia, or admission to oncology service.

**Figure:**
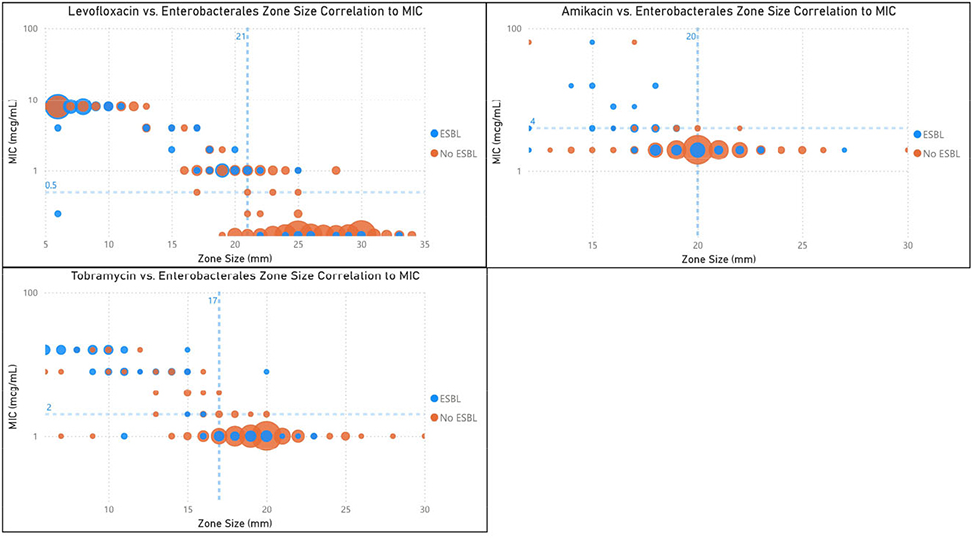
Non-Beta-Lactam Zone size correlation to MIC in Enterobacterales In each graph, a dotted line runs through the y axis at the MIC breakpoint (AST) and a dotted line runs through the x axis at the zone size cutoff (dDD). The upper left quadrant represents the true negative (resistant by dDD and AST) isolates and the bottom right quadrant represents the true positives (susceptible by dDD and AST). The top right quadrant represents the very major errors (susceptible by dDD and resistant by AST) while the bottom left quadrant represents the major errors (resistant by dDD and susceptible by AST).

**Figure:**
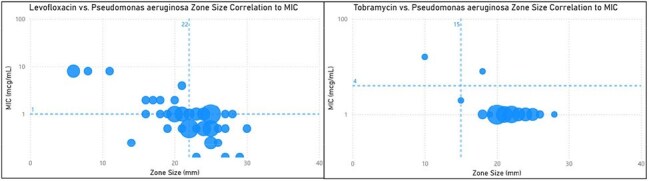
Non-Beta-Lactam Zone size correlation to MIC in Pseudomonas aeruginosa In each graph, a dotted line runs through the y axis at the MIC breakpoint (AST) and a dotted line runs through the x axis at the zone size cutoff (dDD). The upper left quadrant represents the true negative (resistant by dDD and AST) isolates and the bottom right quadrant represents the true positives (susceptible by dDD and AST). The top right quadrant represents the very major errors (susceptible by dDD and resistant by AST) while the bottom left quadrant represents the major errors (resistant by dDD and susceptible by AST).

**Results:**

2826 patients were found with gram negative bacteremia, with 617 remaining for analyses after exclusions (Enterobacterales n=487, Pseudomonas aeruginosa n=70). There is a high PPV for beta-lactam antibiotics tested against *Pseudomonas aeruginosa* (94-98%) and Enterobacterales (93-100%) using dDD results. The NPV of the test was widely variable for Enterobacterales (2-99%) but was slightly more reliable in Pseudomonas aeruginosa (40-100%). The test was much more likely to overcall resistance than to undercall resistance. Few very major errors were identified in both *Enterobacterales (n=33)* and *Pseudomonas aeruginosa (n=6)*.

**Conclusion:**

The reliability of dDD to detect susceptibility in *Enterobacterales* and *Pseudomonas aeruginosa* is highly accurate as demonstrated by the PPV for each antibiotic tested. Our study provides supportive evidence for the utility of dDD to provide quicker actionable results to escalate therapy when an active intervention is coupled with the result. Further study is needed on whether these results actually improve time to appropriate therapy.

**Disclosures:**

Kathryn DeSear, PharmD, BCIDP, Abbvie: Advisor/Consultant|Biomerieux: Advisor/Consultant|Cormedix: Speaking|GSK: Advisor/Consultant|Shionogi: Case based discussion

